# Levodopa-Reduced *Mucuna pruriens* Seed Extract Shows Neuroprotective Effects against Parkinson’s Disease in Murine Microglia and Human Neuroblastoma Cells, *Caenorhabditis elegans*, and *Drosophila melanogaster*

**DOI:** 10.3390/nu10091139

**Published:** 2018-08-22

**Authors:** Shelby L. Johnson, Hyun Y. Park, Nicholas A. DaSilva, Dhiraj A. Vattem, Hang Ma, Navindra P. Seeram

**Affiliations:** 1 School of Biotechnology and Health Sciences, Wuyi University; International Healthcare Innovation Institute (Jiangmen), Jiangmen 529020, China; shelby_johnson@uri.edu; 2 Bioactive Botanical Research Laboratory, Department of Biomedical and Pharmaceutical Sciences, College of Pharmacy, University of Rhode Island, Kingston, RI 02881, USA; ndasilva@my.uri.edu; 3 George and Anne Ryan Institute for Neuroscience, University of Rhode Island, Kingston, RI 02881, USA; 4 Edison Biotechnology Institute, Ohio University, Athens, OH 45701, USA; parkh4@ohio.edu; 5 School of Applied Health Sciences and Wellness, Ohio University, Athens, OH 45701, USA

**Keywords:** *Mucuna pruriens*, levodopa, Parkinson’s disease, neuroprotection, *Caenorhabditis elegans*, *Drosophila melanogaster*

## Abstract

*Mucuna pruriens* (Mucuna) has been prescribed in Ayurveda for various brain ailments including ‘kampavata’ (tremors) or Parkinson’s disease (PD). While Mucuna is a well-known natural source of levodopa (L-dopa), published studies suggest that other bioactive compounds may also be responsible for its anti-PD effects. To investigate this hypothesis, an L-dopa reduced (<0.1%) *M. pruriens* seeds extract (MPE) was prepared and evaluated for its anti-PD effects in cellular (murine BV-2 microglia and human SH-SY5Y neuroblastoma cells), *Caenorhabditis elegans*, and *Drosophila melanogaster* models. In BV-2 cells, MPE (12.5–50 μg/mL) reduced hydrogen peroxide-induced cytotoxicity (15.7−18.6%), decreased reactive oxygen species production (29.1−61.6%), and lowered lipopolysaccharide (LPS)-induced nitric oxide species release by 8.9–60%. MPE (12.5−50 μg/mL) mitigated SH-SY5Y cell apoptosis by 6.9−40.0% in a non-contact co-culture assay with cell-free supernatants from LPS-treated BV-2 cells. MPE (12.5−50 μg/mL) reduced 6-hydroxydopamine (6-OHDA)-induced cell death of SH-SY5Y cells by 11.85–38.5%. Furthermore, MPE (12.5−50 μg/mL) increased median (25%) and maximum survival (47.8%) of *C. elegans* exposed to the dopaminergic neurotoxin, methyl-4-phenylpyridinium. MPE (40 μg/mL) ameliorated dopaminergic neurotoxin (6-OHDA and rotenone) induced precipitation of innate negative geotaxis behavior of *D. melanogaster* by 35.3 and 32.8%, respectively. Therefore, MPE contains bioactive compounds, beyond L-dopa, which may impart neuroprotective effects against PD.

## 1. Introduction

Parkinson’s disease (PD) is a progressive neurodegenerative disease that leads to impaired motor function and is characterized by a loss of dopaminergic neurons in the substantia nigra and is second only to Alzheimer’s disease in its prevalence [1]. The etiology and pathophysiology of PD are not very well understood and have consequently stifled the development of effective therapeutic interventions for PD. Accumulating evidence suggests that elevated oxidative stress and neuroinflammation associated with microgliosis and intracellular aggregation of α-synuclein molecules may be responsible for dopaminergic neuronal atrophy and ultimately the clinical manifestation of PD [2,3,4].

*Mucuna pruriens*, commonly known as Mucuna or velvet bean, is native to eastern India and western regions of China. Mucuna seeds, a rich source of naturally occurring levodopa (L-dopa; 4–7% in Mucuna seeds) [5], have been used traditionally as an effective remedy for several brain related maladies, including reducing tremors (as seen in PD), as documented in the ancient treatise of Ayurveda, the Indian traditional system of medicine [6]. The lack of effective pharmaceutical treatments has stimulated research interest in Mucuna as a PD therapeutic agent in several animal studies and a limited number of human clinical trials [7,8,9]. For example, Mucuna, at a dosage of 17.5 mg/kg, improved motor function and reduced dyskinesia in patients with advanced PD with fewer adverse effects as compared with the conventional treatment of L-dopa paired with a dopamine decarboxylase inhibitor, namely Carbidopa [9]. Mucuna has also been reported to show protective effects against PD in rodent models by increasing the activity of brain mitochondrial complex-I [10] and reducing motor dysfunction [11,12]. While several studies have attributed the anti-PD activities to naturally occurring high levels of L-dopa in Mucuna, emerging evidence suggests that other bioactive compounds besides L-dopa may also have neuroprotective effects. For example, a Mucuna methanolic extract (0.1% dosage) containing low levels of L-dopa (0.01%) showed anti-PD effects including improvements of motor function and olfactory response in a *Drosophila melanogaster* genetic model of PD [13]. The anti-PD effects of the Mucuna methanolic extract were superior to that of the treatment of L-dopa (0.01%) alone in the aforementioned *D. melanogaster* model, suggesting that the overall anti-PD effects of Mucuna were a result of other compounds beyond L-dopa alone [13].

Our group has previously reported on the development of a neuroprotective potential algorithm for several Ayurvedic botanical extracts, among which *M. pruriens* ranked in the top four [14]. Given our group’s research interest in this medicinal plant, and to explore the role of its ‘non-L-dopa’ bioactives against PD, we prepared a *M. pruriens* seed extract (MPE) containing low amounts of L-dopa (<0.1%) with the following objectives: (1) to evaluate the antioxidant and anti-inflammatory effects of MPE in murine microglia (BV-2) and human neuroblastoma (SH-SY5Y) cells; (2) to assess the neuroprotective effects of MPE against neurotoxin-induced cytotoxicity in cellular PD models; and (3) to evaluate the neuroprotective effects of MPE using *Caenorhabditis elegans* and *D. melanogaster* models of chemically induced PD.

## 2. Materials and Methods

### 2.1. Chemicals

Dimethylsulfoxide (DMSO), levodopa (L-dopa), Resveratrol (Resv), lipopolysaccharide (LPS), 2′,7′-dichlorofluorescin diacetate (DCF-DA), hydrogen peroxide (H_2_O_2_), 6-hydroxydopamine (6-OHDA), 1-methyl-4-phenylpyridinium (MPP^+^), and rotenone were purchased from Sigma-Aldrich Chemical Co. (St. Louis, MO, USA). Dulbecco’s modified Eagle’s medium (DMEM)/F-12, phenol red-free DMEM medium and trypsin-versene were purchased from Life Technologies (Grand Island, NY, USA).

### 2.2. Preparation of Mucuna pruriens Seeds Extract (MPE)

*Mucuna pruriens* seeds (3–7% L-dopa) were botanically authenticated and generously provided by Verdure Sciences (Noblesville, IN, USA). *Mucuna pruriens* seeds were authenticated by Dr. V. Singh (Pharmanza, Gujarat, India) with voucher specimen (No. PHPL/HB/013) deposited in the Heber-Youngken Garden and Greenhouse at the College of Pharmacy, the University of Rhode Island, RI, USA. Briefly, the ground *M. pruriens* seeds (150 g) were extracted with sonication in methanol (1000 mL) in an ultrasonic bath (Bransonic 8510; Branson Ultrasonics Corp., Danbury, CT, USA) for 0.5 h and macerated in methanol at room temperature for 24 h to afford a crude methanol extract (6.5 g), which was dried in vacuo (in a water bath at 35 °C); reconstituted in water; and then partitioned sequentially in *n*-hexanes, ethyl acetate, and butanol with details as follows. The dried crude extract (6.5 g) was reconstituted in distilled water (250 mL) and sequentially partitioned with *n*-hexanes, ethyl acetate, and butanol (250 mL × 3 for each solvent). Each of these fractions, namely, hexanes (0.2 g), ethyl acetate (0.3 g), butanol (2.9 g), and the remaining water portion (3.0 g), were dried in vacuo (in a water bath at 35 °C) to afford respective extracts. The levels of L-dopa were quantified in each dried extract (described below) and the extract with the lowest level of L-dopa, namely, MPE (the *M. pruriens* ethyl acetate extract; see Table 1), was selected for further biological evaluation.

### 2.3. Quantification of L-Dopa by Liquid Chromatography Electrospray Ionization Tandem Mass Spectrometry (LC-ESI-MS/MS)

L-dopa was quantified by liquid chromatography electrospray ionization tandem mass spectrometry (LC-ESI–MS/MS) using methods and parameters published by our group and others with some modifications [15,16,17,18,19]. L-dopa quantifications were performed on a prominence ultra-fast liquid chromatography (UFLC) system (Shimadzu, Marlborough, MA, USA) coupled with a QTRAP 4500 system (Applied Biosystems/MDS Sciex, Framingham, MA, USA) with data acquired using Analyst 1.6.3 software and processed using MultiQuant 3.0.1 software (Sciex, Framingham, MA, USA). The UFLC system consisted of three LC-20AD pumps, a DGU-20A degassing unit, SIL-20AC auto sampler, CTO-20AC column oven, and CBM-20A communication bus module. Chromatographic separation was performed using a 100 mm × 4.6 mm i.d., 5 μm, XBridge C18 column (Waters, Milford, MA, USA). The mobile phase consisted of A (water containing 0.1% (*v*/*v*) formic acid) and B (methanol containing 0.1% (*v*/*v*) formic acid) with a gradient elution of 1% B from 0 to 10 min, and 1−4% B from 10 to 20 min. The flow rate was 0.5 mL/min and the injection volume was 10 μL. The column temperature was maintained at 40 °C. The MS operated in electrospray ionization (ESI) in positive mode with multiple reaction monitoring (MRM). Nitrogen was used as the source gas in all cases. Parameters were optimized as follows: IonSpray voltage, 4500 V; nebulizer gas, 40 psi; auxiliary heater gas, 45 psi; curtain gas, 20 psi; turbo gas temperature, 300 °C. Using an authentic L-dopa standard (purchased from Sigma-Aldrich Chemical Co.; St. Louis, MO, USA), L-dopa was analyzed by the multiple reaction monitor (MRM) mode using ion transition at *m*/*z* values of 198/152. All of the analyses of the standard and extracts were performed in triplicates (see LC-ESI-MS/MS spectra in the Supplementary Materials; Figure S1). The calibration curve (*y* = 5006.29*x* − 13189.13; *R* = 0.99825) was acquired by plotting the peak area against the nominal concentrations of L-dopa. The linearity was in the range of 10–1000 ng/mL. The presence of L-dopa in Mucuna extracts was identified as a peak with a retention time of 3.95 min under the ion transition 198/152. The percentage of L-dopa in the different Mucuna extracts was calculated as follows: (ng/mL of L-dopa in extract)/(μg/mL of extract injected) × 100%.

### 2.4. Cell Culture

Murine microglia (BV-2) cells were kindly provided by Dr. Grace Y. Sun (University of Missouri at Columbia, MO, USA) and human neuroblastoma (SH-SY5Y) cells were purchased from American Type Culture Collection (ATCC, Manassas, VA, USA). Cells were maintained at 37 °C in 5% CO_2_ with high glucose (4.5 g/L) DMEM/F-12 accompanied with 10% heat inactivated fetal bovine serum, and 1% P/S (100 U/mL penicillin, 100 mg/mL streptomycin) (Life Technologies, Gaithersburg, MD, USA). MPE was dissolved in distilled water to obtain a 10 mg/mL stock solution and further diluted in serum free media for treatments. Resv (used as a positive control for the cellular based assays) was dissolved in DMSO (10 mM) and diluted in media to the desired concentration. Control cells were treated with 0.1% DMSO in serum free media.

### 2.5. Cell Viability

BV-2 and SH-SY5Y cells were seeded in white walled 96-well plates at 1 × 10^5^ cells/mL in serum free media. MPE (12.5, 25, and 50 μg/mL) were evaluated for cytotoxicity effects in BV-2 and SH-SY5Y cells. After 24 h, cell viability was determined using Cell Titer Glo 2.0 (CTG; Promega, Madison, WI, USA) according to methods previously reported by our group [14,20]. MPE was then evaluated for its cellular protective effects against several oxidative insults as follows. Cells were pretreated with MPE (12.5, 25, and 50 μg/mL), Resv (20 μM), or solvent control (0.1% DMSO) for either 1 h (in BV-2 cells) or 2 h (in SH-SY5Y cells). Cellular oxidative stress was induced in BV-2 and SH-SY5Y with H_2_O_2_ (100 μM), SH-SY5Y with 6-OHDA (25 μM), and MPP^+^ (2 mM). Cellular viability of BV-2 and SH-SY5Y cells after treatment were determined at 6 and 24 h, respectively, by the aforementioned CTG assay.

### 2.6. Determination of Hydrogen Peroxide (H_2_O_2_)-Induced Reactive Oxygen Species (ROS) in Murine Microglia BV-2 Cells

The production of H_2_O_2_-induced reactive oxygen species (ROS) in BV-2 cells was determined by a fluorescent probe (DCF-DA) using previously reported method with modifications [21]. BV-2 microglial cells were seeded in a black 96-well plate at 1 × 10^5^ cells/mL in serum free media. Cells were allowed to attach for 24 h and pretreated with MPE (12.5, 25, and 50 μg/mL), Resv (20 μM), or solvent control (0.1% DMSO) for 1 h. Next, DCF-DA (20 μM) was added to each well and incubated for 25 min. Cells were then washed with PBS and incubated with H_2_O_2_ (100 μM) for 6 h. The fluorescence signal of each cell was measured at excitation and emission wavelengths of 495 nm and 529 nm, respectively, using a SpectraMax M2 plate reader (Molecular Devices, Sunnyvale, CA, USA).

### 2.7. Measurement of Lipopolysaccharide (LPS)-Induced Nitric Oxide Species (NOS) in Murine Microglia BV-2 Cells

The production of total nitric oxide species (NOS) was determined using the Griess reagent as previously reported by our group [14,20]. BV-2 cells were seeded in clear 24-well plates at 1 × 10^5^ cells/mL in serum free media. Cells were treated with MPE (12.5, 25, and 50 μg/mL), Resv (20 μM), or solvent control (0.1% DMSO) for 1 h. The cells were exposed to inflammatory stress induced by treating with LPS (1 μg/mL) for 24 h. Next, culture media from each well were transferred to a 96-well plate and measured for total NOS using the Griess reagent kit (Promega, Fitchburg, WI, USA). Absorbance values were recorded using the SpectraMax M2 plate reader (Molecular Devices, Sunnyvale, CA, USA) at 535 nm.

### 2.8. Non-Contact Co-Culture Assay with BV-2 and SH-SY5Y Cells

The non-contact co-culture assay was performed according to protocols previously reported by our group [22]. Briefly, SH-SY5Y cells were seeded in white wall and clear bottom 96-well plates and allowed to adhere for 24 h. BV-2 cells were plated in 24 well plates and treated with MPE (12.5, 25, and 50 μg/mL), Resv (20 μM), or solvent control (0.1% DMSO), followed by LPS (1 μg/mL) treatment for 24 h. Media from each treatment was collected and centrifuged at 15,000 rpm for 10 min. After centrifugation, BV-2 cell supernatant was used to treat SH-SY5Y cells for 24 h. Cellular viability of SH-SY5Y cells was determined using the CTG assay.

### 2.9. 1-Methyl-4-Phenylpyridinium (MPP^+^) Induced Dopaminergic Neurotoxicity in C. elegans

Wild type *C. elegans* (N2) were maintained on nematode growth media culture plates at 20 °C and age synchronized as previously reported by our group [22]. Then, 40 μL of age synchronized L1 worms washed in S-complete were transferred to a 96-well microplate (approximately 20 worms/well) with *Escherichia coli* OP50 (5 mg/mL), MPP^+^ (750 μM), and MPE (20 or 40 μg/mL) to a final volume of 50 μL. S-complete media was used for control groups. Live worms were counted every 12 h post treatment until no live worms remained.

### 2.10. D. melanogaster Strains and Maintenance

Wild type STR-5 flies were obtained from the Bloomington Stock Center (Department of Biology, Indiana University, Bloomington, IN, USA). Strains were reared on Formula 4-24^®^ Instant *Drosophila* Medium (Carolina Biological Supply, Burlington, NC, USA) and reared on Bloomington Formulation (Genesee Scientific, San Diego, CA, USA) at 25 °C with 75% humidity and a 12-hour light/dark cycle [23]. Approximately 40–50 mating pairs were transferred into flasks and allowed to lay eggs. After nine days, newly eclosed male flies were collected over a period of three days and used in further experiments.

### 2.11. Negative Geotaxis (Climbing) Assay in D. melanogaster

Newly eclosed wild type (STR-5) male flies were randomly separated into 10 groups of 50 flies each and transferred to control flasks (media only) or in treatment flasks (media + 40 μg/mL MPE). To induce neurotoxicity, every four days, flies were starved in empty vials for 24 h and transferred into vials containing a filter paper saturated with 1 mL of 10% sucrose (blank), 6-OHDA (1 mM), or rotenone (500 μM). After 24 h, the flies were transferred into vials with a fresh supply of their respective diets and used for climbing assay on day 10 post-eclosion. Flies from control and treatment groups were then tapped into the bottom of graduated cylinder (diameter: 2.7 cm, height: 25 cm) superimposed with a ruler and allowed to climb for 10 s. Flies were photographed (Canon, Inc., Tokyo, Japan, JP, EOS 50D; 15.1 MP Digital SLR) at *t*_0_ and *t*_10_ seconds to calculate the climbing distance [24].

### 2.12. Statistical Analyses

All data are presented as mean ± standard errors of three separate biological samples. Analyses of cellular data were conducted by analysis of variance (ANOVA) followed by Dunnett’s test for multiple comparisons of group means. The Kaplan–Meier method was used to compare the survival curves of *C. elegans* and the survival differences were tested for statistical significance using the log rank test (Mantel Cox). For the *D. melanogaster* climbing assay, Welch’s *t*-test was used to compare the different treatment groups and generate *p* values (alpha = 0.05). Significance compared with control group is presented as *p* ≤ 0.05 (#), *p* ≤ 0.001 (###), and *p* ≤ 0.0001 (####). Significance for all tests compared with toxic treatment was defined as follows: *p* ≤ 0.05 (*), *p* ≤ 0.01 (**), *p* ≤ 0.001 (***), and *p* ≤ 0.0001 (****). GraphPad Prism software 6.0 (GraphPad Software, Inc., San Diego, CA, USA) was used to calculate statistics for both the in vitro and in vivo analyses.

## 3. Results and Discussion

### 3.1. Preparation of Levodopa (L-Dopa)-Reduced Mucuna pruriens Extract (MPE)

*Mucuna pruriens* is a medicinal plant that is well known to naturally contain L-dopa (4–7%) [5], which might be attributed to its neuroprotective effects against PD [6]. However, the presence of other phytochemicals in *M. pruriens*, including polyphenols (tannins, flavonoids, gallic acid, phenolic acids), saponins, terpenoids, alkaloids, and fatty acids, have been reported with various pharmacological activities (see Supplementary Materials; Figure S2 and Table S1) [6,25,26,27,28,29]. Recent studies also suggest that phytochemicals apart from L-dopa may also contribute to the overall neuroprotective activities of *M. pruriens* [13,30]. Therefore, in this study, we prepared a *M. pruriens* seed extract (MPE) containing reduced L-dopa levels (<0.1%), which was subsequently evaluated for its neuroprotective effects using a panel of in vitro and in vivo assays. The seeds of *M. pruriens* were extracted/solvent-solvent partitioned in varying solvents to yield extracts, which were evaluated for L-dopa content by liquid chromatography electrospray ionization tandem mass spectrometry (LC-ESI–MS/MS). As shown in Table 1, the L-dopa levels in the initial methanol *M. pruriens* seeds extract was 28.0%, which was significantly reduced to 0.03% in the ethyl acetate *M. pruriens* extract (MPE). As even this low level (0.03%) of L-dopa could impart biological effects, we evaluated a pure L-dopa solution (<0.1%) in several of the in vitro assays. Our preliminary data showed that the MPE, but not this pure L-dopa was active in these assays (data shown in Supplementary Materials Figures S5 and S6). Therefore, this MPE extract was selected for further evaluation of its neuroprotective effects in a panel of cell-based and in vivo bioassays as described below.

### 3.2. MPE Reduces Hydrogen Peroxide (H_2_O_2_)-Induced Toxicity and Reactive Oxygen Species (ROS) Production in Microglia BV-2 Cells

**Figure 1:**
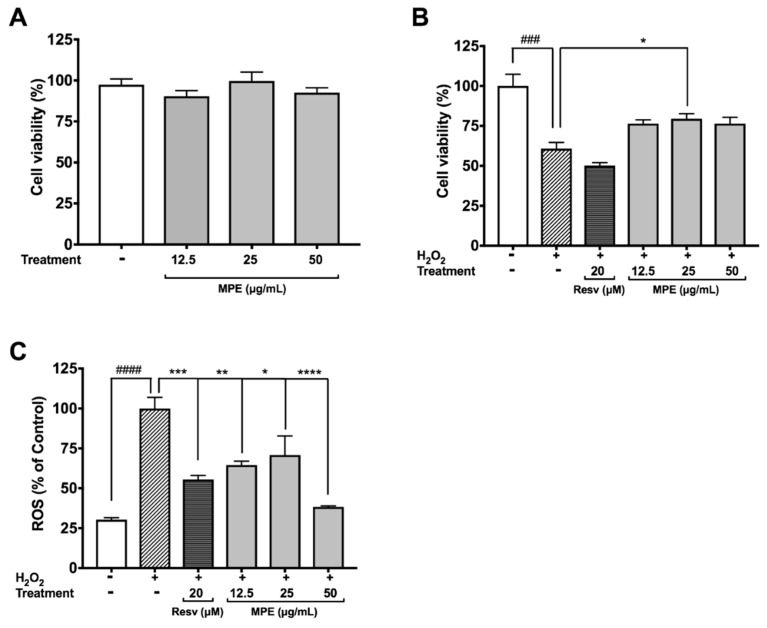
Effects of *Mucuna Pruriens* Seeds Extract (MPE) (12.5, 25, 50 μg/mL) on cellular viability and reactive oxygen species (ROS) levels in BV-2 cells. Effects on BV-2 cellular viability by MPE alone (**A**); by MPE after H_2_O_2_-induced BV-2 cell toxicity (**B**); and on ROS levels after BV-2 cell exposure to H_2_O_2_ (**C**). All data expressed as mean ± standard error (*n* = 3), significance was reported by analysis of variance (ANOVA) followed with Dunnett multiple comparison testing, as compared with control *p* ≤ 0.001 (###), and *p* ≤ 0.0001 (####); as compared with toxic agent, *p* ≤ 0.05 (*), *p* ≤ 0.01 (**), *p* ≤ 0.001 (***), and *p* ≤ 0.0001 (****). Resv—Resveratrol.

Microglia are the native immune cells of the central nervous system (CNS) that undergo activation and proliferation to carry out phagocytosis, release inflammatory cytokines, and produce ROS and reactive nitrogen species (RNS) in response to injury and/or infection. Unresolved inflammation and excessive oxidant production by microglia are lethal to both neuronal and non-neuronal cells in the CNS and have been associated with PD. All of the Mucuna extracts including the crude methanol, hexanes, ethyl acetate (MPE), butanol, and water extracts (at 25 µg/mL) were evaluated for their protective effects against H_2_O_2_-induced toxicity in BV-2 cells. Our data showed that among the extracts, only the MPE significantly increased the viability of BV-2 cells exposed to H_2_O_2_ (see Supplementary Materials Figure S3A). Therefore, we evaluated the effects of MPE on oxidative stress induced by H_2_O_2_ in microglia BV-2 cells. MPE (12.5, 25, and 50 μg/mL) was non-toxic to BV-2 cells with cell viability greater than 90.3% at 24 h (Figure 1A). As shown in Figure 1B, the cell viability of H_2_O_2_-treated BV-2 cells decreased by 39.2%, as compared with the control group. Although MPE, at concentrations of 12.5, 25, and 50 μg/mL, showed a trend to ameliorate the H_2_O_2_-induced cytotoxicity in BV-2 cells, only MPE at a concentration of 25 μg/mL significantly increased the cell viability of H_2_O_2_-treated BV-2 cells, by 18.6%. The protective effects of MPE against the production of ROS by H_2_O_2_ in BV-2 cells were then evaluated. As shown in Figure 1C, the production of ROS in H_2_O_2_-treated BV-2 cells was elevated by 3.29-fold as compared with the control cells. MPE (12.5, 25, and 50 μg/mL) reduced the H_2_O_2_-induced production of ROS by 35.5, 29.1, and 61.6%, respectively, compared with the H_2_O_2_-treated BV-2 cells. Resveratrol (Resv; 20 μM), used as the positive control, reduced the H_2_O_2_-induced production of ROS by 44.52%. These results are in agreement with our previous observation, wherein an *M. pruriens* water extract increased viabilities of murine BV-2 microglia and differentiated human SH-SY5Y neuronal cells that exposed to H_2_O_2_ [14]. Moreover, studies from other research groups also reported that *Mucuna* seeds powder (300 mg/kg/BW in diet) reduced oxidative stress in rodent sperm cells [31].

### 3.3. MPE Reduces Lipopolysaccharide (LPS)-Induced Nitric Oxide Species (NOS) Production in Microglia BV-2 Cells and Protects SH-SY5Y Cells in a Co-Culture Model

**Figure 2:**
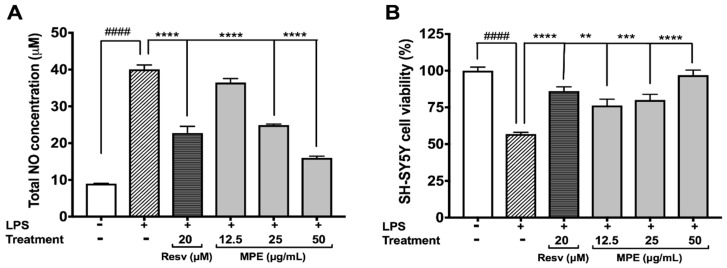
Effects of MPE (12.5, 25, 50 μg/mL) on production of nitric oxide in BV-2 microglia and resulting influence in non-contact co-culture in SH-SY5Y neuroblastoma. Effects on levels of nitric oxide produced in BV-2 induced with lipopolysaccharide (LPS) (**A**), and on SH-SY5Y cell viability after co-culture with BV-2 LPS-induced media (**B**). All data expressed as mean ± standard error (*n* = 3), significance was reported by ANOVA followed with Dunnett multiple comparison testing, as compared with control *p* ≤ 0.0001 (####); as compared with toxic agent, *p* ≤ 0.01 (**), *p* ≤ 0.001 (***), and *p* ≤ 0.0001 (****).

Elevated production of NOS leading to massive neuronal death has been implicated in PD [32]. All of the aforementioned Mucuna extracts (at 25 µg/mL) were evaluated for their protective effects against LPS-induced NO production in BV-2 cells. Among the extracts, MPE showed the highest ability to reduce NO production in BV-2 cells exposed to LPS (see Supplementary Materials Figure S3B). Therefore, MPE was evaluated for its protective effects against neuroinflammation induced by LPS in BV-2 cells and in a non-contact co-culture model with SH-SY5Y neuroblastoma cells [22]. As shown in Figure 2A, LPS increased the NOS production in BV-2 cells by 4.46-fold as compared with the control group (control 8.987 μM vs. LPS 40.06 μM). MPE (12.5, 25, and 50 μg/mL) reduced the NOS production in LPS-stimulated BV-2 cells by 8.9, 37.8, and 60.1%, respectively, as compared with the cells treated with LPS alone. Resv (positive control; 20 μM), also reduced the NOS production by 43.2% in the LPS-treated BV-2 cells and was similar to our previous observation [22]. In the non-contact co-culture model (Figure 2B), conditioned media collected from BV-2 cells treated with LPS alone reduced the cell viability of SH-SY5Y cells by 43.1%. The conditioned media from treatment of LPS and MPE (12.5, 25 and 50 μg/mL) significantly increased the cellular viability of SH-SY5Y cells by 19.4, 23.2%, and 40.1%, respectively, as compared with the cells treated with media from LPS-treated BV-2 cells. The positive control, Resv (20 μM), also increased the cell viability of SH-SY5Y cells by 29.3%. Our results support other studies on Mucuna reporting a reduction in nitrite levels induced by 1-methyl-4-phenyl-1,2,3,6-tetrahydropyridine (MPTP) in the nigrostriatal region of Parkinsonian mice brain [33]. 

### 3.4. MPE Reduces Oxidative Stress Induced Cytotoxicity in SH-SY5Y Cells

**Figure 3:**
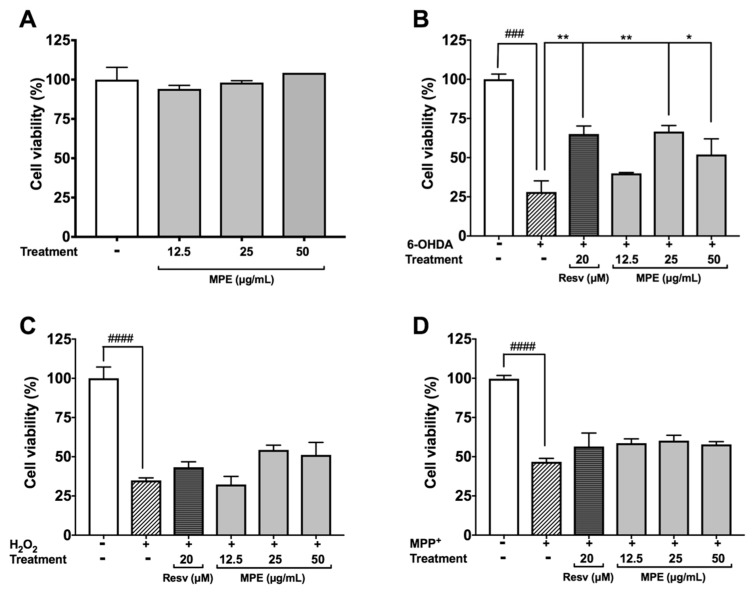
Effects of MPE (12.5, 25, and 50 μg/mL) on cellular viability of SH-SY5Y human neuroblastoma cells against toxic models of Parkinson’s disease. Effects of MPE (12.5, 25, and 50 μg/mL) alone on SH-SY5Y cell viability (**A**), of MPE after 6-OHDA-induced toxicity (**B**), of MPE against H_2_O_2_-induced toxicity (**C**), and of MPE against MPP^+^ induced toxicity (**D**). Data shown as mean ± standard error (*n* = 3), significance was reported by ANOVA and subsequent Dunnett multiple as compared with control *p* ≤ 0.001 (###), and *p* ≤ 0.0001 (####); as compared with toxic agent, *p* ≤ 0.05 (*), and *p* ≤ 0.01 (**).

Several neurotoxins, including 6-OHDA and MPP, induce oxidative cytotoxicity in dopaminergic neurons by multiple mechanisms and thus are used to model PD [2,34,35]. The protective effects of MPE were evaluated in SH-SY5Y neuroblastoma cells against oxidative stress induced neurotoxicity. MPE (12.5, 25, and 50 μg/mL) did not induce cytotoxicity of SH-SY5Y cells after 24 h incubation (viability >90%) in the CTG assay (Figure 3A). Toxicity was induced in SH-SY5Y neuroblastoma by treatment with 6-OHDA, H_2_O_2_, and MPP^+^ (25 μM, 100 μM, and 2 mM, respectively). Treatment of 6-OHDA significantly reduced the viability of SH-SY5Y cells by 71.9% as compared with the control group, while MPE (12.5, 25, and 50 μg/mL) reduced 6-OHDA-induced cell death of SH-SY5Y cells by increasing cell viability compared with the 6-OHDA treated group by 11.9%, 38.5%, and 23.9%, respectively (Figure 3B). Treatment of H_2_O_2_ reduced SH-SY5Y cells viability by 65.1% as compared with control, while MPE (at higher concentrations of 25 and 50 μg/mL) showed moderate protective effects by increasing SH-SY5Y cell viability compared with cell viability of the H_2_O_2_ treated group by 19.5% and 16.3%, respectively (Figure 3C). MPP^+^ treatment significantly reduced the viability of SH-SY5Y cells by 46.7% as compared with control (Figure 3D); however, MPE showed no protective effects. Our findings obtained from these cellular PD models are in agreement with previously reported neuroprotective effects of *Mucuna* in neurotoxins-induced PD animal models. For example, *Mucuna* treatment reduced 6-OHDA-induced L-dopa depletion in nigrostriatal tract of rats with PD symptoms [10,33]. 

### 3.5. MPE Reduces Lethality of MPP^+^ Induced Dopaminergic Neurotoxicity in C. elegans

The neurotoxin, MPTP, is metabolized to MPP^+^ by monoamine oxidase-B and is subsequently taken up by dopaminergic neurons, where it inhibits mitochondrial complex I, resulting in ATP depletion to induce neuronal death [35]. Therefore, we evaluated the effects of MPE against MPP^+^ dopaminergic neurotoxicity in wild type *C. elegans*. The effects of MPE in MPP^+^ induced neurotoxic paralysis and lethality in *C. elegans* were evaluated at concentrations of 20 and 40 μg/mL (IC_10_ = 42.1 μg/mL). The median and maximum survival of worms after exposure to 750 μM MPP^+^ was 72 h (Table 2).

**Figure 4:**
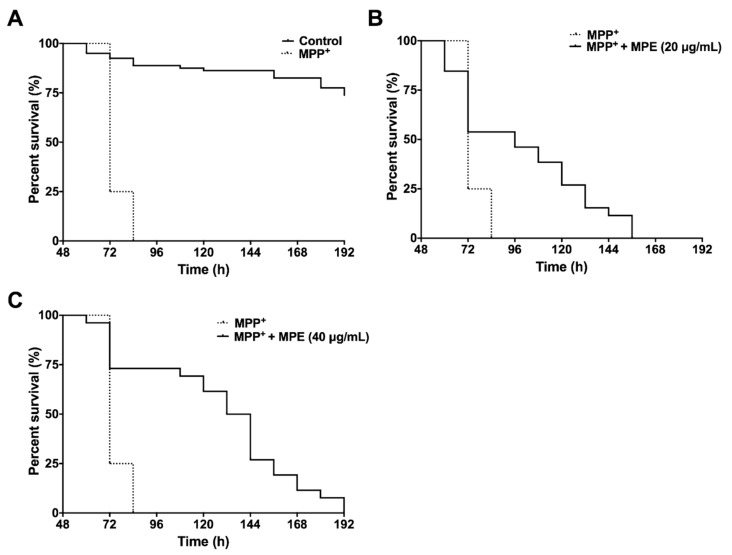
Effects of MPE (20 and 40 μg/mL) on lifespan of *C. elegans* after MPP^+^ exposure. MPP^+^ at concentration of 750 μM reduces *C. elegans* lifespan, as compared with control group (**A**). MPE at concentrations 20 μg/mL (**B**) and 40 μg/mL (**C**) increase *C. elegans* lifespan, as compared with toxic MPP^+^ exposure (750 μM). Survival curves of *C. elegans* were statistically analyzed by log rank test (Mantel Cox), as compared with MPP^+^ treatment (*n* > 100).

Treatment of MPP+ significantly reduced the median and maximum survival by 3.2-fold (72 h) and 3.5-fold (72 h), respectively, compared with worms in the control group (Figure 4A). MPE at 20 μg/mL significantly increased (*p* < 0.001) the median and maximum survival by 1.3-fold (96 h) and 1.9-fold (138 h), respectively, compared with worms treated with MPP^+^ alone (Figure 4B). MPE at 40 μg/mL significantly increased the mean and maximum survival in *C. elegans* by 1.8-fold (132 h) and 2.25-fold (162 h) respectively, compared with worms treated with MPP^+^ alone (Figure 4C and Table 2).

### 3.6. MPE Abrogates Chemically Induced Neurotoxicity in D. melanogaster

Changes in several behavioral phenotypes of *D. melanogaster* in response to genetically or chemically induced neurotoxicity have been exploited extensively to evaluate potential neuroprotective effects of therapeutics [36]. As MPE was significantly more neuroprotective at 40 μg/mL in reducing MPP^+^ induced dopaminergic neurotoxicity in *C. elegans* (Figure 4), we used this dosage to determine its effect on climbing behavior (negative geotaxis) in *D. melanogaster* neurotoxin induced PD model. The two neurotoxins (6-OHDA and rotenone) used in our study induce a PD-like phenotype in *D. melanogaster* characterized by several behavioral changes including a muted innate negative geotaxis response due to locomotor defects. The aforementioned toxins generally injure dopamine neurons and cause behavioral defects including climbing, which can be measured by negative geotaxis assay. Similar to MPP^+^, rotenone is another mitochondrial complex I inhibitor that causes ATP impairment and ROS production, and induces neuronal death [35]. In our study, *D. melanogaster* were exposed to 6-OHDA and rotenone to induce PD like phenotype. After 10 days, flies exposed to neurotoxins showed a highly muted climbing ability compared with control group. This loss of negative geotaxis ability was significantly ameliorated when flies were pre-treated with MPE.

**Figure 5:**
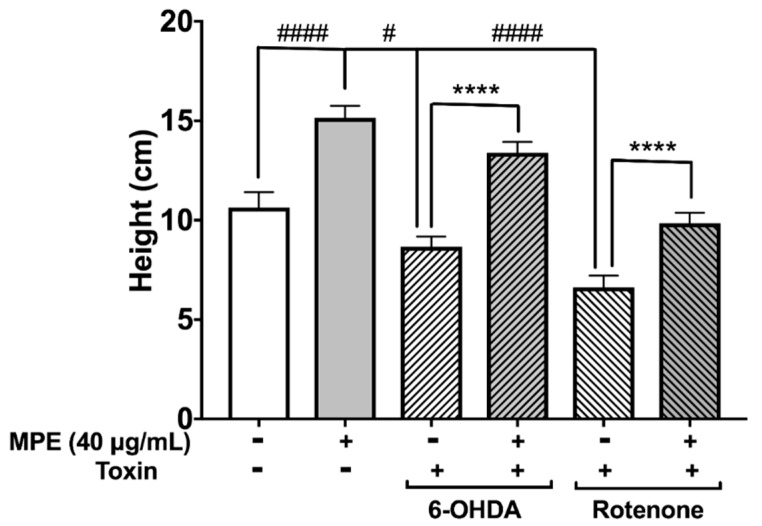
Effects of MPE on negative geotaxis in *Drosophila melanogaster*. Effects of MPE (40 μg/mL) alone on climbing ability, by MPE after 6-OHDA (1 mM) exposure, and on MPE climbing ability after rotenone exposure (500 μM). Significance was determined as compared with control and *p* ≤ 0.05 (#) and *p* ≤ 0.0001 (####); compared with toxic treatment using Welch’s t-test with three replicates of *n* > 40, *p* ≤ 0.0001 (****).

The median climbing distance in 6-OHDA treated flies and rotenone treated flies was 18.6% (8.7 cm; *p* ≤ 0.05) and 37.8% (6.2 cm, *p* ≤ 0.001) lower than in control flies (10.95 cm), respectively (Figure 5). Treatment of MPE alone significantly increased the climbing distance in flies by 42.5% (15.9 cm) compared with the control group (Figure 5). Pre-treatment with MPE abrogated the effect of neurotoxins on climbing behavior. In the MPE + 6-OHDA treated flies, the median climbing distance was 54.5% (13.95 cm) higher as compared with flies that were treated with 6-OHDA alone (Figure 5). In the MPE + rotenone treated flies, this was 48.7% (9.9 cm) higher than in flies that were exposed to rotenone only without any MPE pre-treatment (Figure 5). Our results on the neuroprotective effects of MPE on neurotoxin induced PD models using *C. elegans* and *D. melanogaster* support previous studies with Mucuna in rodent models of PD using MPTP [33] and 6-OHDA [25] and provide further evidence on the neuroprotective effects of non-L-dopa bioactives in MPE.

## 4. Conclusions

In summary, we developed a L-dopa reduced *Mucuna pruriens* extract (MPE) and evaluated its neuroprotective effects in murine microglia BV-2 and neuroblastoma SH-SY5Y cells. MPE treatment decreased BV-2 and SH-SY5Y cytotoxicity induced by oxidative stress and inflammation. In addition, MPE ameliorated dopaminergic neurotoxin-induced lethality in SH-SY5Y (6-OHDA), *C. elegans*, and recovered climbing ability *D. melanogaster* models for PD. Taken the data from the in vitro and in vivo experiments together, MPE showed neuroprotective effects in our PD models. Studies on the anti-PD effects of purified compounds isolated from MPE and their potential mechanism/s of action will be pursued by our group in the future.

## Figures and Tables

**Table 1:** Levodopa (L-dopa) content for each *Mucuna pruriens* seed extracts as determined by liquid chromatography electrospray ionization tandem mass spectrometry (LC-ESI-MS/MS).

Extract	Yield (%; *w*/*w*)	L-Dopa Content (%; *w*/*w*)
methanol	100	28.0
hexanes	3.1	0.54
ethyl acetate	4.7	0.03
butanol	45.3	10.05
water	46.9	21.39

**Table 2:** Survival (median and maximum) of *C. elegans* (N2), as compared with 1-methyl-4-phenylpyridinium (MPP^+^) treatment (750 μM). MPE treatment at both 20 and 40 μg/mL significantly increased survival, as determined by log rank test (Mantel Cox), *n* > 100, *p* ≤ 0.05 (*), *p* ≤ 0.001 (***).

Survival (h)	MPP^+^	MPP^+^ + MPE (20 μg/mL)	MPP^+^ + MPE (40 μg/mL)
Median	72	96 *	138 ***
Maximum	72	132 *	162 ***

## Supplementary Materials

The following are available online at http://www.mdpi.com/2072-6643/10/9/1139/s1, Figure S1: LC-ESI-MS/MS spectra for quantifications of L-dopa in *Mucuna pruriens* extracts, Figure S2: HPLC-DAD chromatograms of Mucuna pruriens extracts, Figure S3: Effects of *Mucuna pruriens* extracts on the cell viability and LPS-induced NO production in murine BV-2 microglia, Figure S4: Morphology of BV-2 murine microglia treated with H2O2 + MPE, H2O2 + 0.07% L-dopa, LPS + MPE, and LPS + 0.07% L-dopa, Figure S5: Effects of MPE and 0.07% L-dopa on H2O2-induced toxicity in murine BV-2 microglia, Figure S6: Effects of MPE and 0.07% L-dopa on LPS-induced NO production in murine BV-2 microglia, Table S1: Chemical constituents of *Mucuna pruriens*.
