# Supplementary material for: Levodopa-Reduced Mucuna pruriens Seed Extract Shows Neuroprotective Effects against Parkinson’s Disease in Murine Microglia and Human Neuroblastoma Cells, Caenorhabditis elegans, and Drosophila melanogaster
Source: Nutrients. 2018 Aug 22;10(9):1139. doi: 10.3390/nu10091139 (PMC6164394; doi:10.3390/nu10091139)
Supplement: Supplementary file 1 [file nutrients-10-01139-s001.pdf]

Supplementary Materials

Levodopa-Reduced *Mucuna pruriens* Seed Extract shows Neuroprotective Effects against Parkinson's Disease in Murine Microglia and Human Neuroblastoma Cells, *Caenorhabditis elegans*, and *Drosophila melanogaster*

Shelby L. Johnson[2,3], Hyun Young Park[4,5], Nicholas A. DaSilva[2,3], Dhiraj A. Vattem[4,5*], Hang Ma[1,2,3*], Navindra P. Seeram[2,3*]

[1] School of Biotechnology and Health Sciences, Wuyi University; International Healthcare Innovation Institute (Jiangmen), Jiangmen 529020, Guangdong, China; hang_ma@uri.edu (H.M.)
[2] Bioactive Botanical Research Laboratory, Department of Biomedical and Pharmaceutical Sciences, College of Pharmacy, University of Rhode Island, Kingston, RI 02881, USA; shelby_johnson@uri.edu (S.L.J.); ndasilva@my.uri.edu (N.A.D.); nseeram@uri.edu (N.P.S.)
[3] George and Anne Ryan Institute for Neuroscience, University of Rhode Island, Kingston, RI 02881, USA
[4] Edison Biotechnology Institute, Ohio University, Athens, OH 45701, USA; vattem@ohio.edu (D.A.V.); parkh4@ohio.edu (H.Y.P.)
[5] School of Applied Health Sciences and Wellness, Ohio University, Athens, OH 45701, USA

List of contents

HPLC-DAD analyses of *Mucuna pruriens* extracts …………………………………………………………3

Morphological analysis………………………………………………………………………………………3

Figure S1. LC-ESI-MS/MS spectra for quantifications of L-dopa in *Mucuna pruriens* extracts ……………...4

Figure S2. HPLC-DAD chromatograms of *Mucuna pruriens* extracts …………………………………….....7

Figure S3. Effects of *Mucuna pruriens* extracts on the cell viability and LPS-induced NO production in murine BV-2 microglia …………………………………………………………………………………...8

Figure S4. Morphology of BV-2 murine microglia treated with H_2O_2+MPE, H_2O_2+0.07% L-dopa, LPS+MPE, and LPS+0.07% L-dopa ………………………………………………………………………….....9

Figure S5. Effects of MPE and 0.07% L-dopa on H_2O_2-induced toxicity in murine BV-2 microglia ……....10

Figure S6. Effects of MPE and 0.07% L-dopa on LPS-induced NO production in murine BV-2 microglia 11

Table S1. Chemical constituents of *Mucuna pruriens* ………………………………………………….....12

References……………………………………………………………………………………………….....13

HPLC-DAD analyses of *Mucuna pruriens* extracts

Chemical profiles of *Mucuna pruriens* extracts were performed using HPLC-DAD method. *M. pruriens* extracts including crude methanol, n-hexanes, ethyl acetate, butanol, and water extracts were prepared in 50% methanol/water (25 mg/mL). The column used was a Waters Sunfire® C18 column (250 mm × 4.6 mm i.d., 5 µm; Milford, MA, USA) at room temperature. Solvent system consisted of 0.1% trifluoroacetic acid in water (A) and methanol (B). 0-25 min 97% A, 25-66 min 50% A, 66-81 min 5% A, 81-95 min 5% A, 95-96 min 97% A, and 96-110 min 97%A at a fow rate of 0.75 mL/min. Wavelength range for DAD detection was 220-520 nm and peaks were monitored at the wavelength of 250 nm. HPLC-DAD chromatograms are shown in Figure S2.

Morphological analyses

Murine BV-2 microglia cells were stained with crystal violet staining post treatments to visualize morphological changes. Cells were fixed in 70% ethanol for 5 min, then stained with 0.5% crystal violet stain (Sigma-Aldrich Chemical Co.,St. Louis, MO, USA) for 10 min. Cells were then washed in phosphate buffered saline, then imaged with EVOS ® FL Cell Imaging System (ThermoFisher Scientific, Waltham, MA, USA) in phase at 40X (Figure S4).

Figure S1. LC-ESI-MS/MS spectra of L-dopa (**A**) and L-dopa in the *Mucuna pruriens* extracts including crude methanol (**B**), n-hexanes (**C**), ethyl acetate (**D**), butanol (**E**), and water (**F**) extracts. The presence of L-dopa in the Mucuna extracts was identified as a peak with a retention time of 3.95 min with an ion transition of 198/152.

(A)

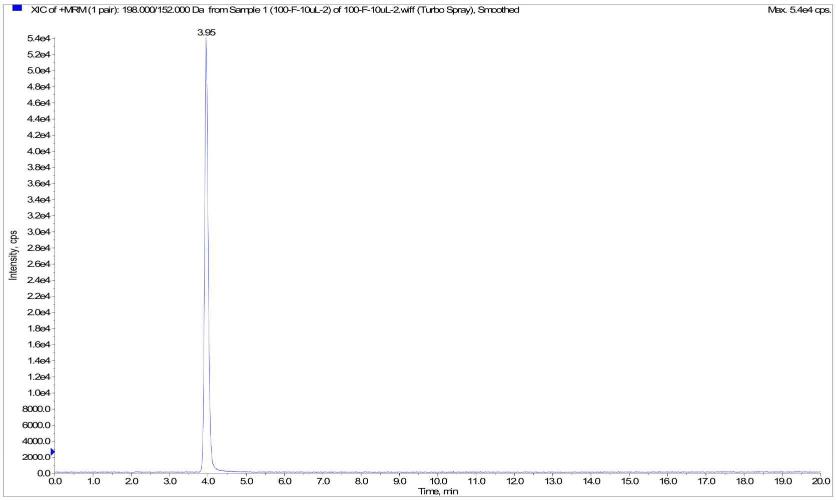

(B)

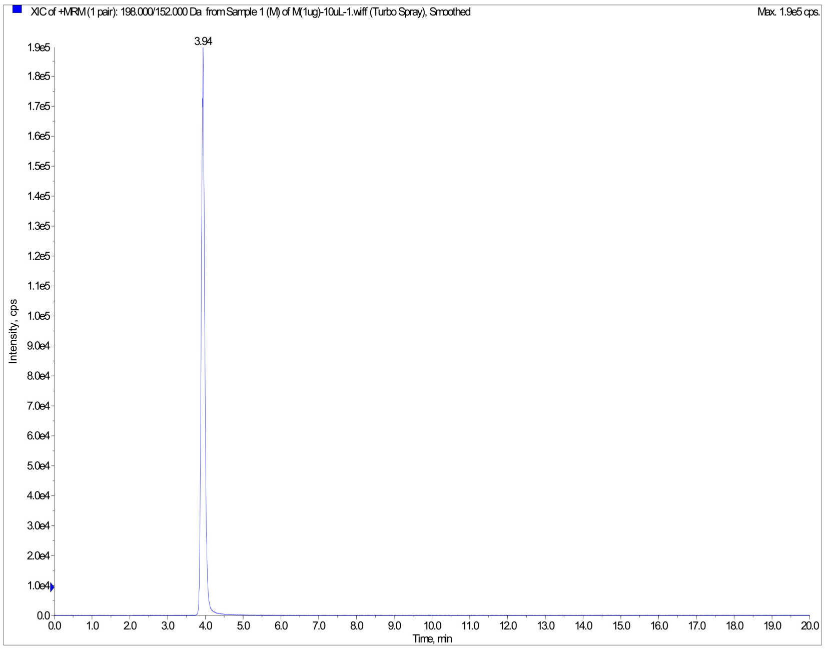

(C)

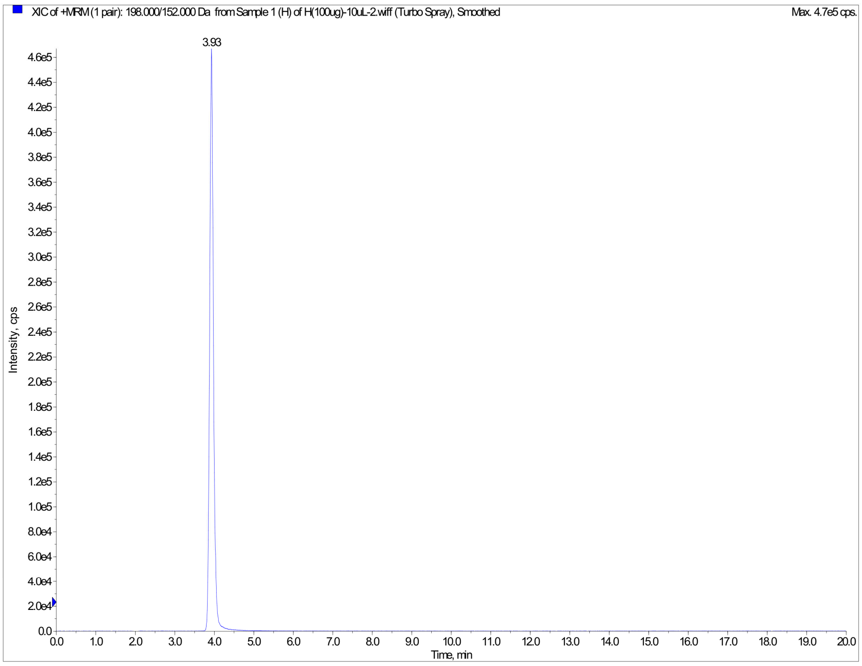
XIC of +MRM (1 pair): 198.000/152.000 Da from Sample 1 (H) of H(100ug)-10uL-2.wiff (Turbo Spray), Smoothed
Max. 4.7e5 cps.
3.93
Intensity, cps
Time, min

(D)

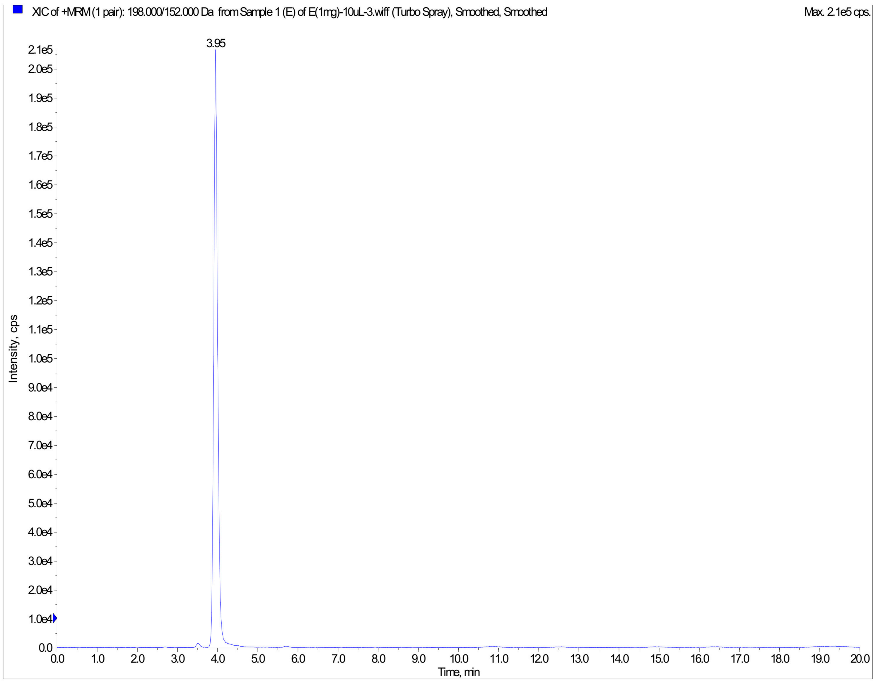
XIC of +MRM (1 pair): 198.000/152.000 Da from Sample 1 (E) of E(1mg)-10uL-3.wiff (Turbo Spray), Smoothed, Smoothed
Max. 2.1e5 cps.
3.95
Intensity, cps
Time, min

(E)

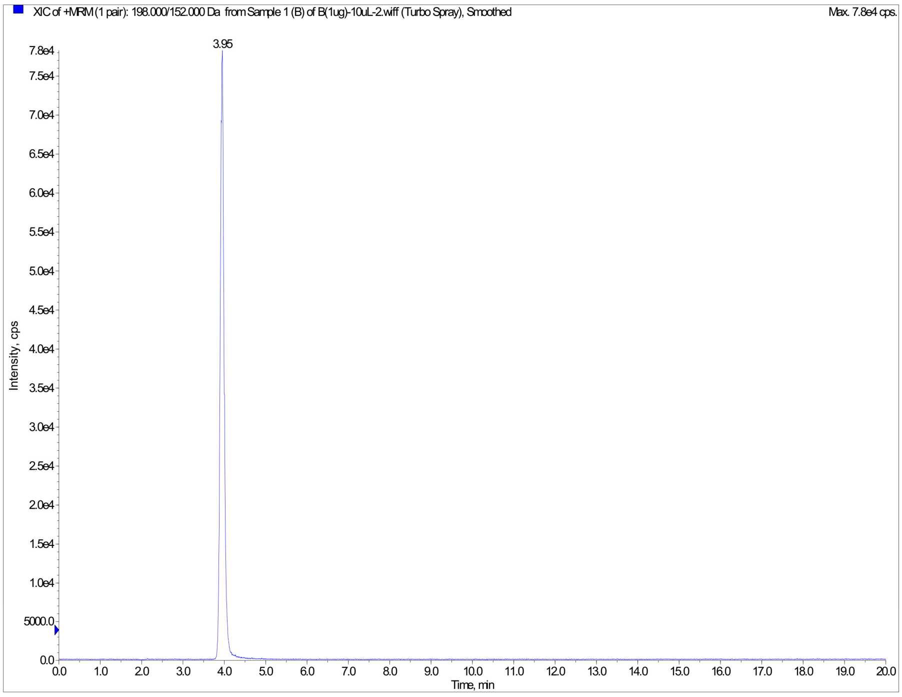

XIC of +MRM (1 pair): 198.000/152.000 Da from Sample 1 (B) of B(1ug)-10uL-2.wiff (Turbo Spray), Smoothed
Max. 7.8e4 cps.
3.95
Intensity, cps
Time, min

(F)

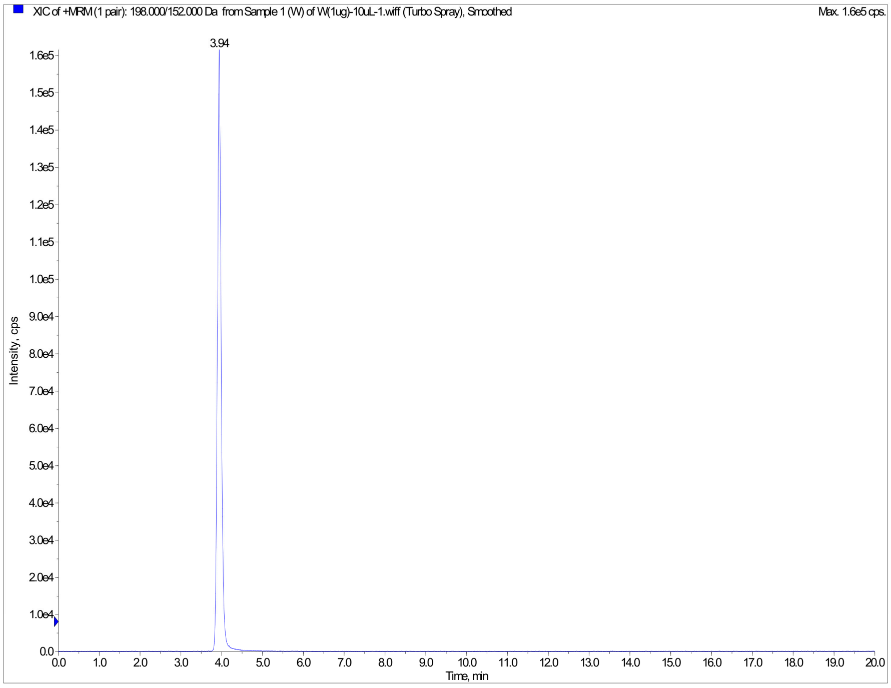

XIC of +MRM (1 pair): 198.000/152.000 Da from Sample 1 (W) of W(1ug)-10uL-1.wiff (Turbo Spray), Smoothed
Max. 1.6e5 cps.
3.94
Intensity, cps
Time, min

Figure S2. HPLC-DAD chromatograms of profiles of *Mucuna pruriens* extracts including crude methanol (**A**), n-hexanes (**B**), ethyl acetate (**C**), butanol (**D**), and water (**E**) extracts. Peaks were monitored at a wavelength of 250 nm.

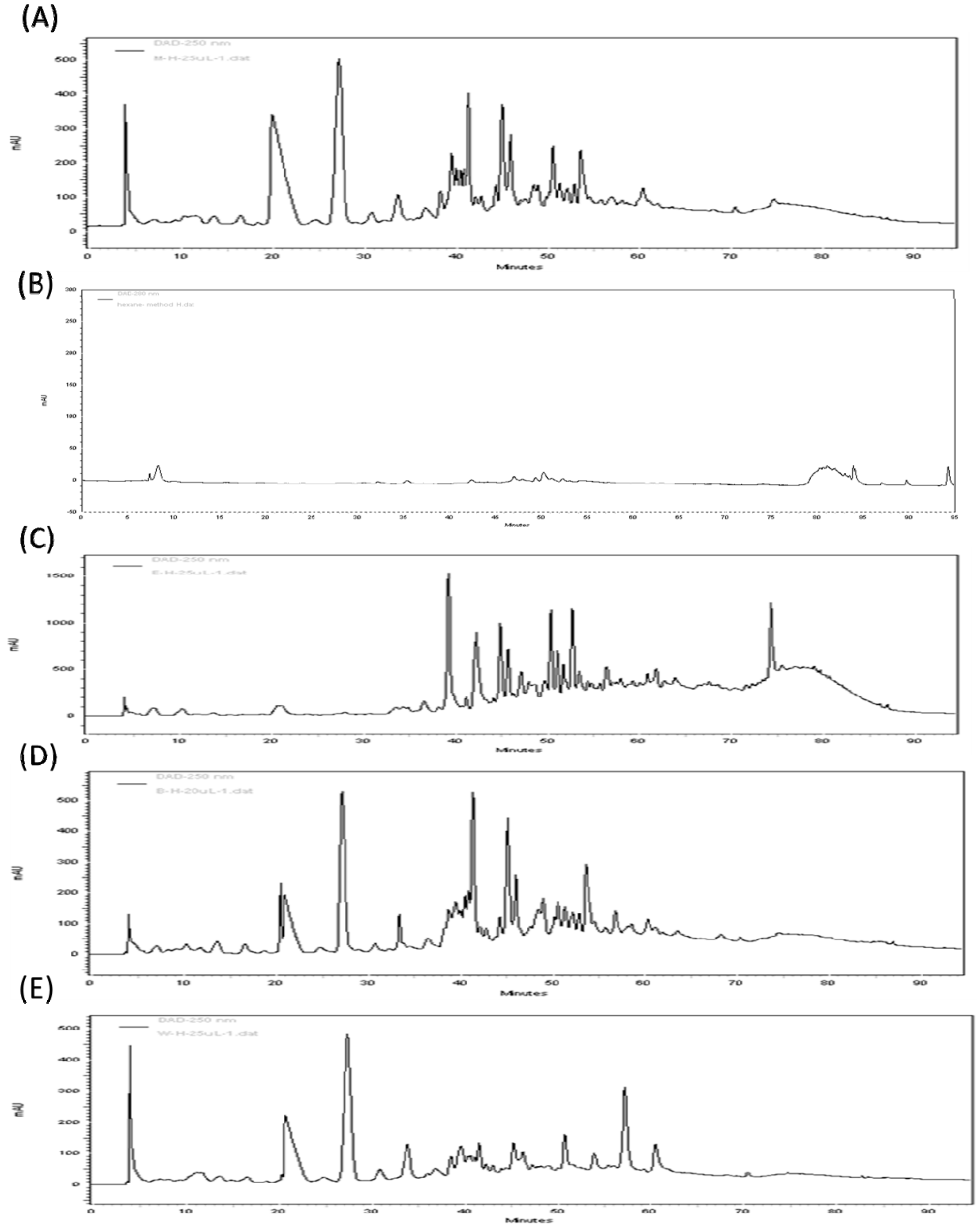

Figure S3. Effects of *Mucuna pruriens* extracts including crude methanol, n-hexanes, ethyl acetate, butanol, and water extract (at concentration of 25 µg/mL) on the cell viability (**A**) and LPS-induced NO production in murine BV-2 microglia (**B**)**.** Significance was reported by ANOVA followed with Dunnett multiple comparison testing, as compared to control p≤0.0001 (####); as compared to toxic agent, p≤0.05 (*), p≤0.001 (***) and p≤0.0001 (****).

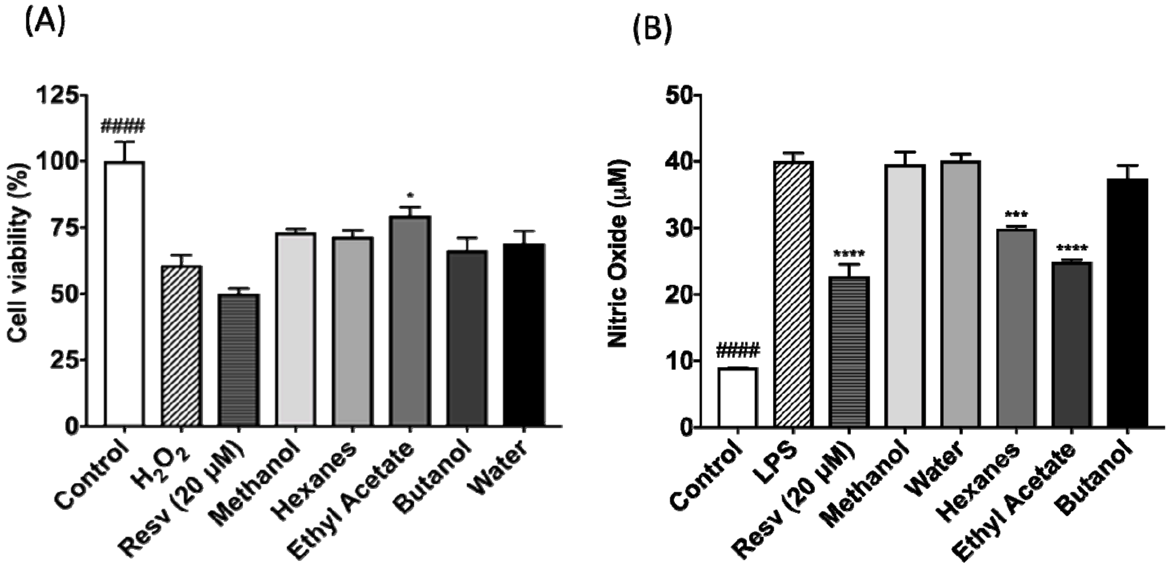

Figure S4. Morphology of murine BV-2 microglia treated with vehicle (**A**), H_2O_2 alone (**B**), H_2O_2+0.07% L-dopa (**C**), and H_2O_2+MPE (**D**); murine BV-2 microglia treated with vehicle (**E**), LPS alone (**F**), LPS+0.07% L-dopa (**G**), and LPS+MPE (**H**).

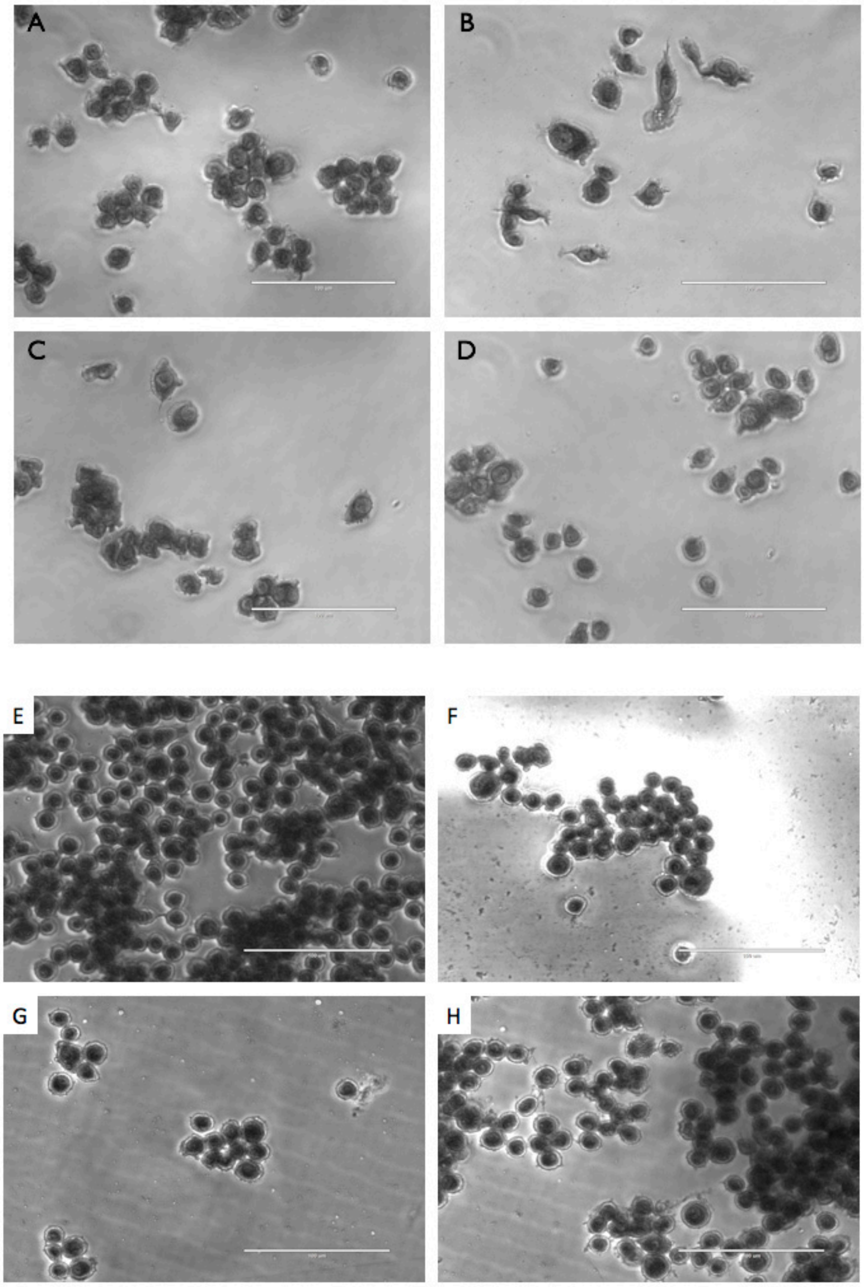

Figure S5. Effects of MPE and 0.07% L-dopa on H_2O_2-induced toxicity in murine BV-2 microglia. Significance was reported by ANOVA followed with Dunnett multiple comparison testing, as compared to control p≤0.0001 (####); as compared to toxic agent, p≤0.05 (*).

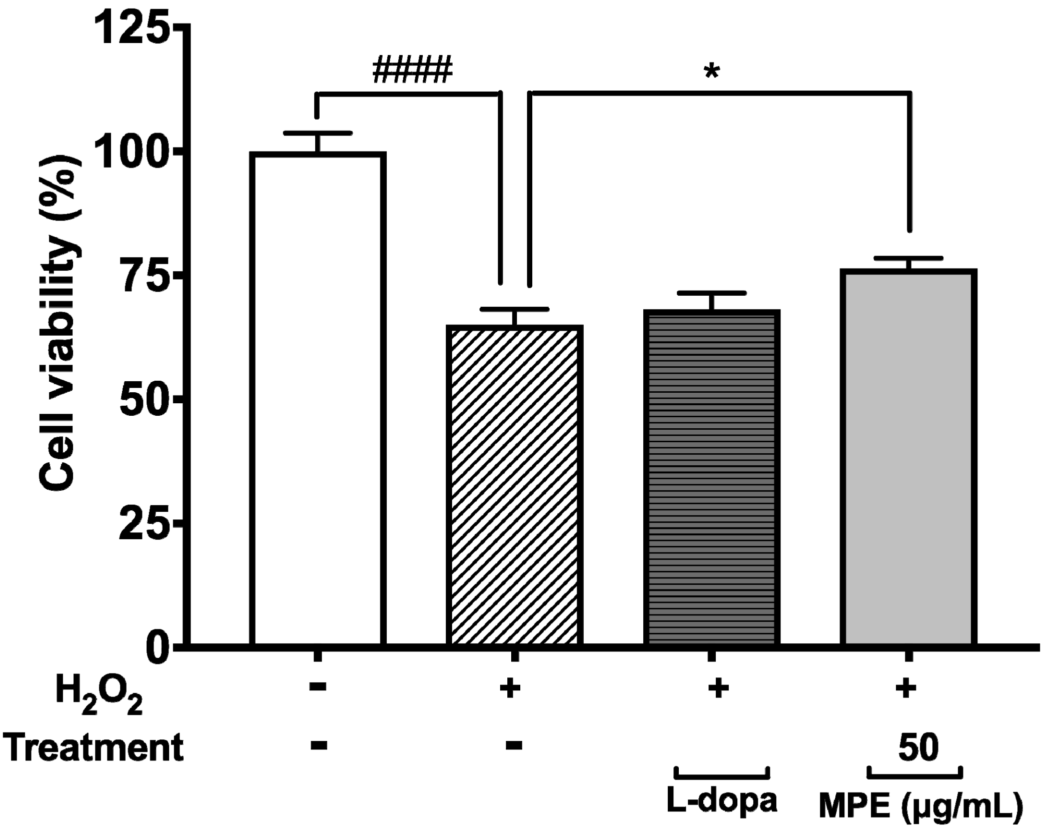

Figure S6. Effects of MPE and 0.07% L-dopa on LPS-induced NO production in murine BV-2 microglia. Significance was reported by ANOVA followed with Dunnett multiple comparison testing, as compared to control p≤0.0001 (####); as compared to toxic agent, p≤0.0001 (****).

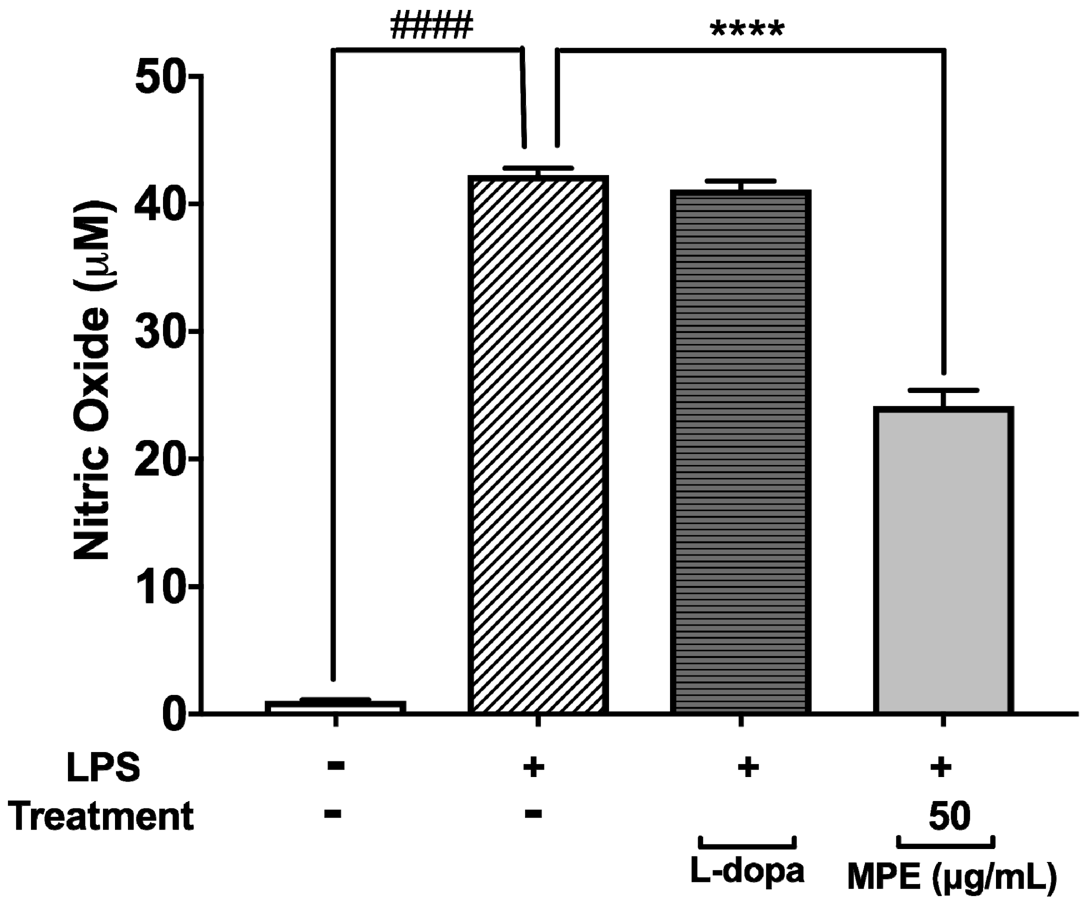

Table S1. Chemical constituents of *Mucuna pruriens*.

Type of chemicals	chemicals	References
Polyphenols	Tannins, flavonoids (e.g. genistein and daidzein), gallic acid, phenolic acids	[1][2]
Saponins		[1][2]
Terpenoids		[1][2]
Alkaloids and amino acids	β-Carboline, N,N-Dimethyl tryptamine, 5- hydroxytryptamine, bufotenine, tetrahydroisoquinoline, hydroisoquinoline, 5-oxyindole- 3- alkylamine, 6- methoxyharman, arahidicacid, arginine, glutathione, indole- 3- alkylamine	[3] [4]
Fatty acids	Linoleic acid, myristic acid, oleic acid, palmitic acid, vernolic acid, stearic acid	[5]
Carbohydrates	oligosaccharides (e.g. raffinose, stachyose, verbascose)	[6]

References:

1. Kasture, S.; Mohan, M.; Kasture, V. *Mucuna pruriens* seeds in treatment of Parkinson's disease: Pharmacological review. *Orient. Pharm. Exp. Med.* **2013**, *13*, 165–174, doi:10.1007/s13596-013-0126-2.

2. Sridhar, K. R.; Bhat, R. Agrobotanical, nutritional and bioactive potential of unconventional legume - Mucuna. *Livest. Res. Rural Dev.* **2007**, *19*.

3. Damodaran, M.; Ramaswamy, R. Isolation of l -3:4-dihydroxyphenylalanine from the seeds of *Mucuna pruriens*. *Biochem. J.* **1937**, *31*, 2149–2152, doi:10.1042/bj0312149.

4. Misra, L.; Wagner, H. Lipid derivatives from *Mucuna pruriens* seeds. *Indian J. Chem. - Sect. B Org. Med. Chem.* **2006**, *45*, 801–804.

5. Natarajan, K.; Narayanan, N.; Ravichandran, N. Review on "Mucuna" - The wonder plant. *Int. J. Pharm. Sci. Rev. Res.* **2012**, *17*, 86–93.

6. Janardhanan, K.; Gurumoorthi, P.; Pugalenthi, M.; Nutritional nutritional potential of five accessions of a south Indian tribal pulse, *Mucuna pruriens* var utilis I. The effect of processing methods on the content of L-Dopa, phytic acid, and oligosaccharides. *Trop. Subtrop. Agroecosystems* **2003**, *1*, 141–152, doi:10.1016/j.theriogenology.2015.06.019.
